# Determining interaction directionality in complex biochemical networks from stationary measurements

**DOI:** 10.1038/s41598-025-86332-0

**Published:** 2025-01-23

**Authors:** N. Leibovich

**Affiliations:** National Research Council of Canada, NRC-Fields Mathematical Sciences Collaboration Centre, 222 College st., Toronto, ON M5T 3J1 Canada

**Keywords:** Computational biology and bioinformatics, Systems biology

## Abstract

Revealing interactions in complex systems from observed collective dynamics constitutes a fundamental inverse problem in science. Some methods may reveal undirected network topology, e.g., using node-node correlation. Yet, the direction of the interaction, thus a causal inference, remains to be determined - especially in steady-state observations. We introduce a method to infer the directionality within this network only from a “snapshot” of the abundances of the relevant molecules. We examine the validity of the approach for different properties of the system and the data recorded, such as the molecule’s level variability, the effect of sampling and measurement errors. Simulations suggest that the given approach successfully infer the reaction rates in various cases.

## Introduction

Many complex systems in physics and biology constitute networks of dynamically interacting units^1^. In these systems, especially in biological ones, the process is often described with stochastic variables^2–4^, and their collective dynamics and functions are governed by the network structure. Determining the underlying interaction topology is essential, for example, for understanding and controlling their function, identifying new pathways in gene regulatory networks or understanding some metabolic mechanisms^5–7^. Particularly, understanding the direction of metabolic reactions is fundamental for predicting cellular behavior, optimizing metabolic engineering strategies, and identifying potential drug targets. Revealing these interaction networks poses a great challenge. Therefore, various studies have examined methods to find the structure of interaction networks^8–13^.

Commonly, interaction networks are constructed from data obtained by time-series measurements, or even pseudo-time trajectories^14–23^. To do so, researchers use some mathematical and statistical tools such as Bayesian inference and maximum likelihood together with machine-learning algorithms^17–22^. There, the collective dynamics are used for network inference—whether the interactions are approximately linear or remain nonlinear, where the latter requires further assumptions on the system^24–27^. However, multidimensional time-dependent synchronized recorded data is not available in some scenarios.

Generally, many biological processes are presumed to reach a steady state^21,27–33^. Importantly, while the population of molecules within a cell or organism is dynamic - constantly undergoing synthesis and degradation - the molecular abundances display time-independent characteristics. The use of steady-state data for the inference of interaction networks has been examined, yet the dynamics are assumed to be linear in the activity of the nodes of the network^34^, or the system is assumed to be externally driven in a controlled way^27,29^. Nevertheless, real systems are not required to fulfill these requirements.

In many cases the collective dynamics evolve via the (Markovian-) Master equation, where stochastic variables possess integer values^35^. Specifically, one of the well known models studied using the Master equation is the stochastic birth-death process which describes stochastic biochemical reactions^35^. In general, interaction networks of variables which evolve with Master dynamics are not limited to biochemical reaction networks, and has also been studied in other disciplines, see^36^ and references therein.

Yet, for consistency, this manuscript is oriented to bio-chemical reaction network studies, with adequate terminology.

In biochemical reaction network studies, one may infer the *functional* connectivity which gains insight into statistical dependencies that the entire set of interactions yield between pairs of units through the collective network dynamics^37,38^. The statistical dependencies between components may be given by the well-known Pearson correlation, the mutual information, or their analyses with silencing methods^38–40^. These, however, are symmetric metrics and as such they are blind to the direction of the interaction providing only non-directed networks. Non-symmetric metrics, such as partial correlations and dependency analyses, require recorded data from all other interacting variables within the network^31,41–44^. That data is not available in many systems.

**Fig. 1:**
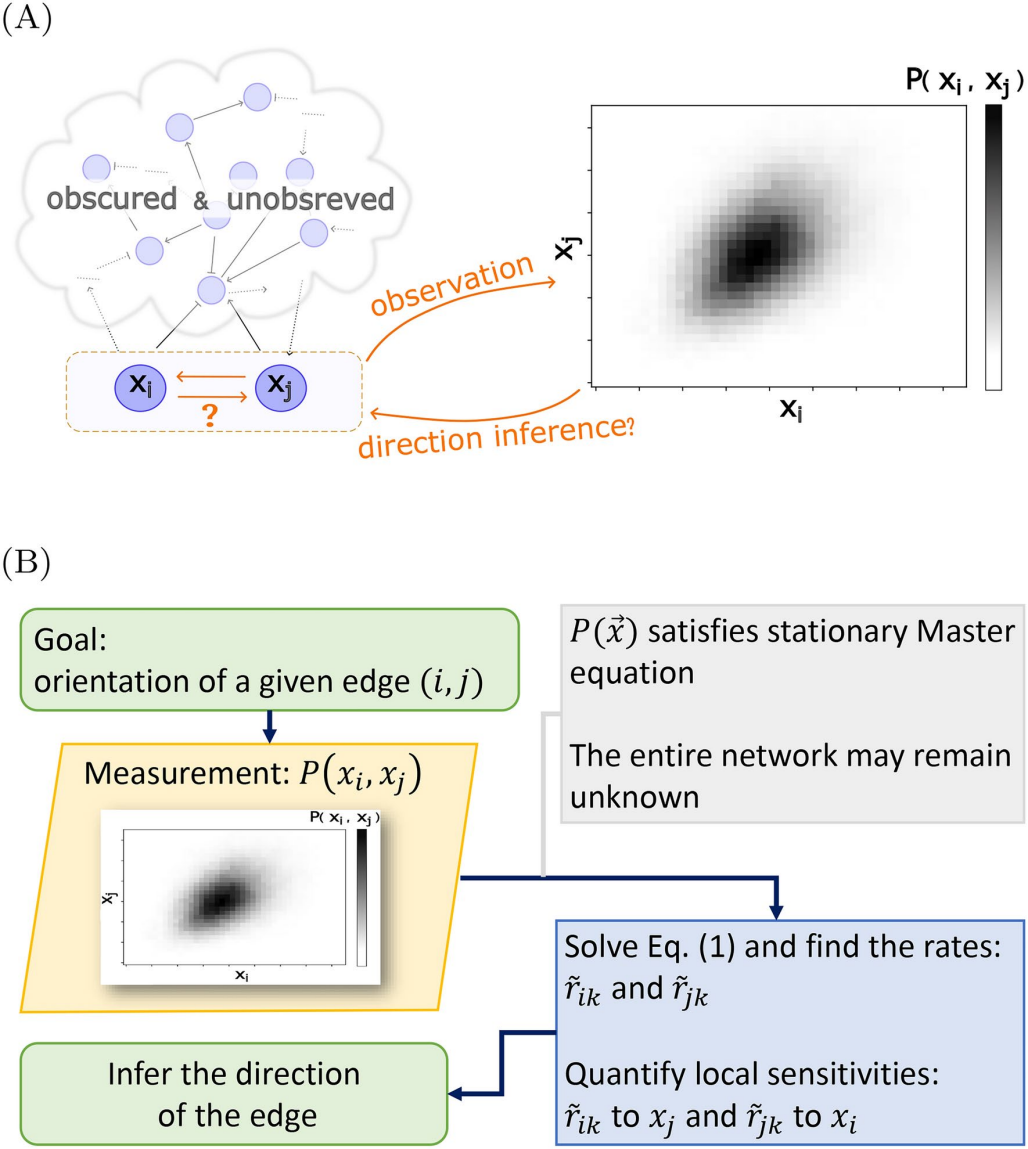
Main goal and inference method flowchart. (**A**) Determining the direction of interaction between stochastic variables is challenged by the lack of temporal observations, further knowledge of the entire network topology, or records of other variables within that network. (**B**) Our method exploits the steady-state joint distribution to infer the direction of arrows using the global balance equation Eq. (1) and the local sensitivity analysis.

Here, we introduce a novel data analysis approach to infer the interaction direction, which under some conditions may be interpreted as causality. Its key advantage is that only the variables under investigation need to be measured, while the mechanistic details of regulation or degradation of all other components within the network do not need to be modeled or even to be observed, see Fig. 1. This approach exploits the global probability flux-balance equation that must be satisfied in the steady state^45–48^. In contrast to existing works, our approach does not require temporal information, experimental perturbations, or complete observation of all components within an interaction network.

## Results

### Theoretical background and overview

Consider *V* variables coupled via an interaction network, i.e., a directed graph with *V* vertices. The variables state vector is given by $\vec {x} =\{x_1, x_2, \dots , x_V\}$, where $x_i$ represents the number of units from type *i*, e.g. its expression or activity level. Each variable $x_i(t) \in {{\mathbb {N}}}_0$ and evolves with time by probabilistic events $x_i \xrightarrow {r_{ik}(\vec {x})} x_i + d_{ik}$ where the *k*-th reaction changes the abundance of the *i*-th molecule $x_i$ by $d_{ik}$. The reaction rates $r_{ik}(\vec {x})$ depend on the state vector $\vec {x}$ according to the interaction network. The stationary Master equation yields the global balance relation, which means that for every node *i*

1 $$\begin{aligned} 0=  \sum _k \langle {\widetilde{r}}_{ik}(x_i -d_{ik}, x_j)|x_i -d_{ik}\rangle { P}(x_i -d_{ik})  -\langle {\widetilde{r}}_{ik}(x_i, x_j)|x_i \rangle { P}(x_i ), \end{aligned}$$

where ${\widetilde{r}}_{ik}(x_i,x_j) \equiv \langle r_{ik} (\vec {x}) |x_i, x_j \rangle$ and the angular brackets represent conditional means. The above relation is derived from a summation of the Master equation over all other variables where consider stationarity $\partial _t P =0$^45–48^, see further derivation in “Methods” and SI.

From Eq. (1) one may obtain the reaction rate only from a given joint steady-state distribution of the two molecules $P(x_i, x_j)$, see “Methods” and^48^. Our approach is based on *local* sensitivity of ${\widetilde{r}}_{ik}$ to $x_j$; a direction of the interaction is inferred, which means an arrow is drawn in the directed graph, if a sufficient sensitivity is quantified, see details in “Methods” and SI. We remark that the suggested directional inference approach solely uses information about $(x_i, x_j)$ , thus the directionality inference problem decomposes over pairs of nodes within the network such that the direction of each interaction can be reconstructed independently.

To evaluate the quality of the inference, we initially consider a class of random networks with Michaelis–Menten kinetics which model gene regulatory networks^49^, and random networks where each unit is a Goodwin oscillator, a prototypical biological oscillator, that characterizes various biological processes such as circadian clocks^50,51^. Additionally, we examine our inference strategy on a biological realistic system based on a subset of an *E. coli * gene regulatory network given in^52–55^. In the following, we evaluate the performance of the suggested approach to identifying the direction of a given interaction.

Importantly, Eq. (1) is not an approximation but an exact flux balance relation at stationarity. However, a finite number of recorded points *N* introduces sampling errors into the distribution $P(\vec {x})$, as shown in SI. These sampling errors may affect the inference of directions. Therefore, we also examine below the impact of the number of sampling points, *N*, on the overall results.

### Performance in general synthetic networks

**Fig. 2:**
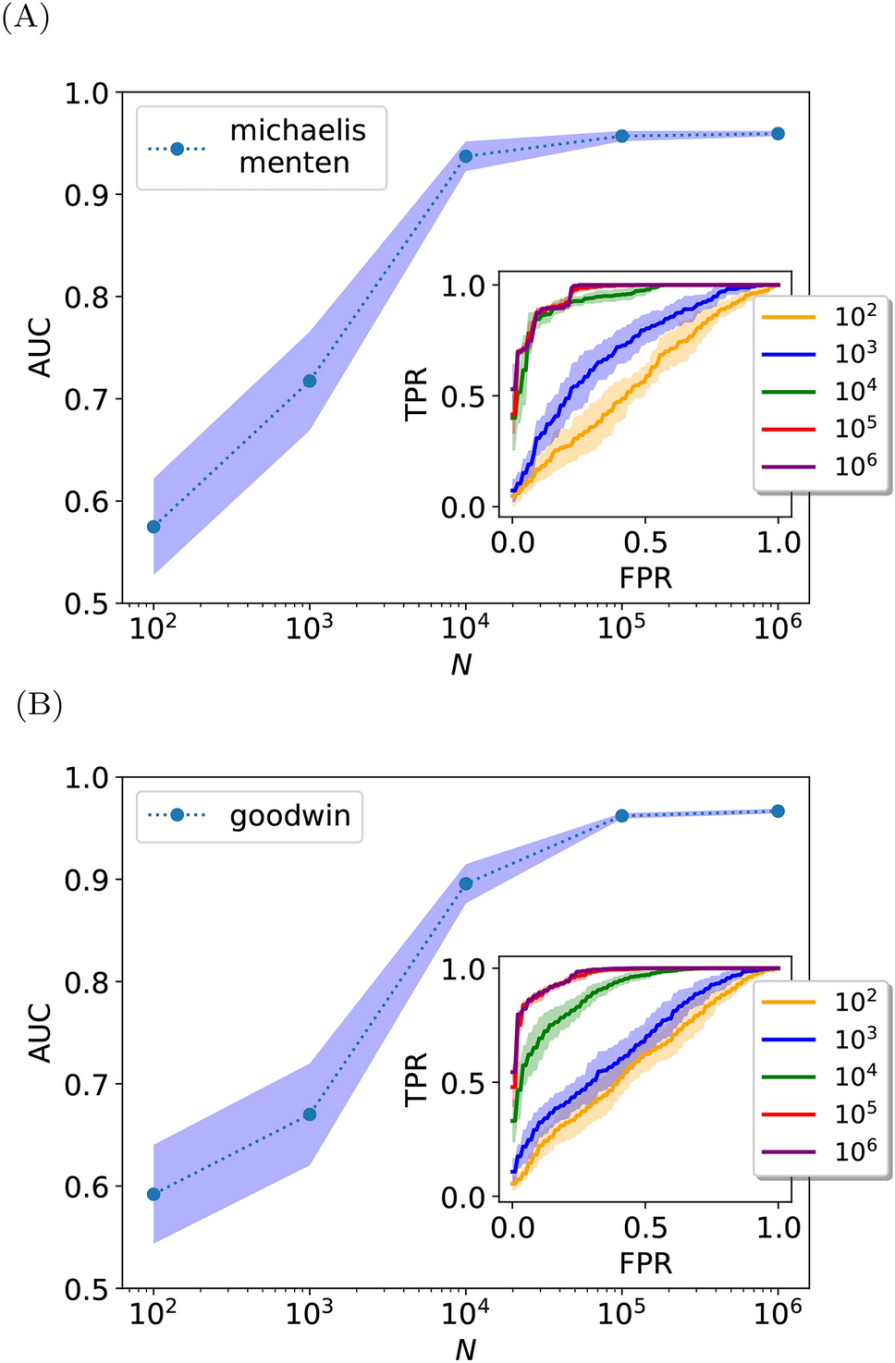
Inferring interaction directions from steady state. Simulation results indicate an improvement with increasing the number of data points *N*. Panel (**A**): The AUC scores for the Michaelis–Menten model (see “Methods”). Inset: its corresponding ROC curves. Panel (**B**): Similar results are obtained for the coupled Goodwin oscillators. The shaded area corresponds to the standard deviation.

**Fig. 3:**
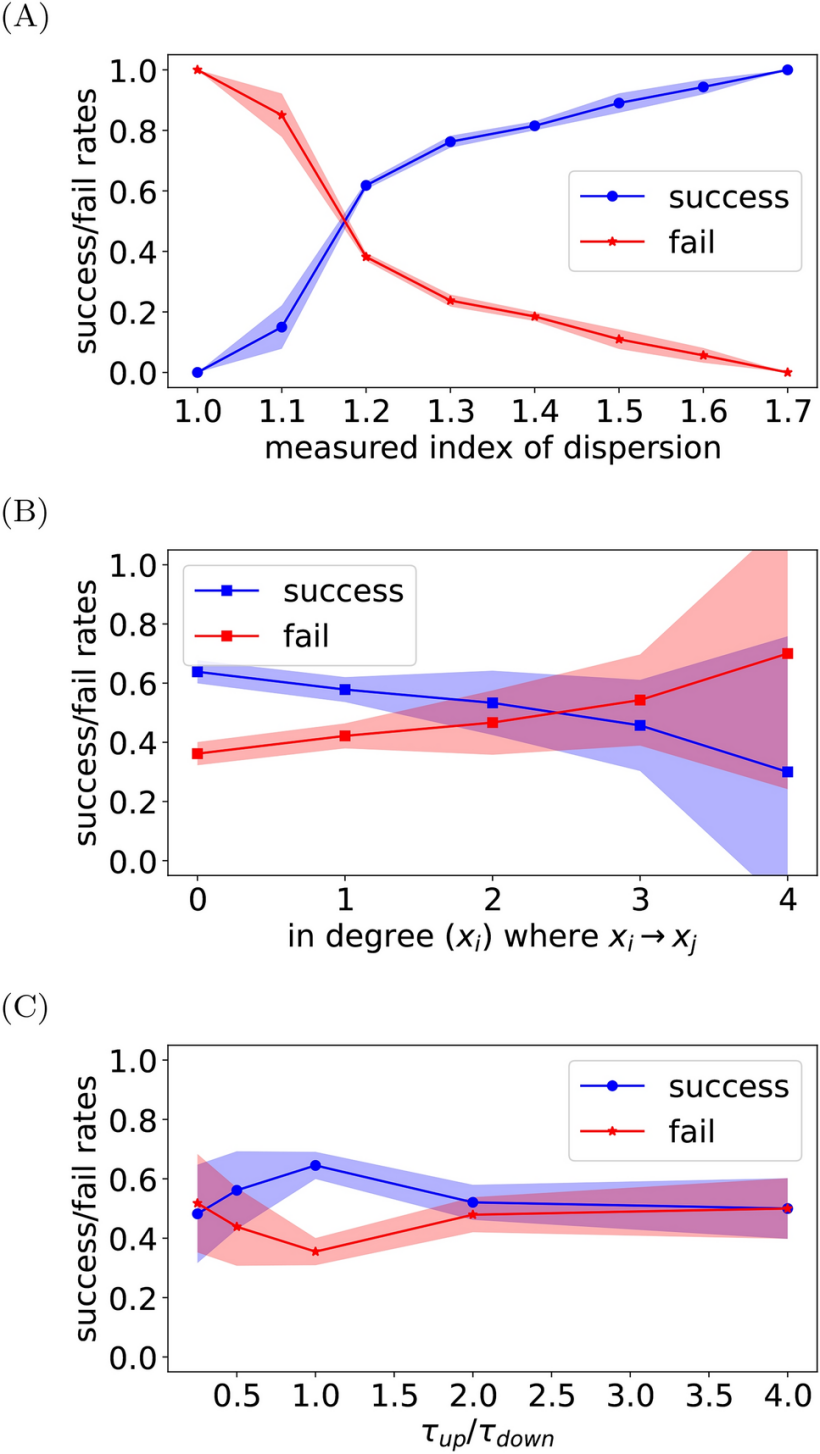
Factors dictate the performance. (**A**): the index of dispersion affects the probability of a successful inference of the direction. Results obtained the Michaelis–Menten model with $N=10^2$. (**B**) Dependence of the degree on the directionality inference and (**C**) lifetimes dependence obtained from the Michaelis–Menten model with $N=10^2$. For all panels the shaded area represents the standard deviation.

**Fig. 4:**
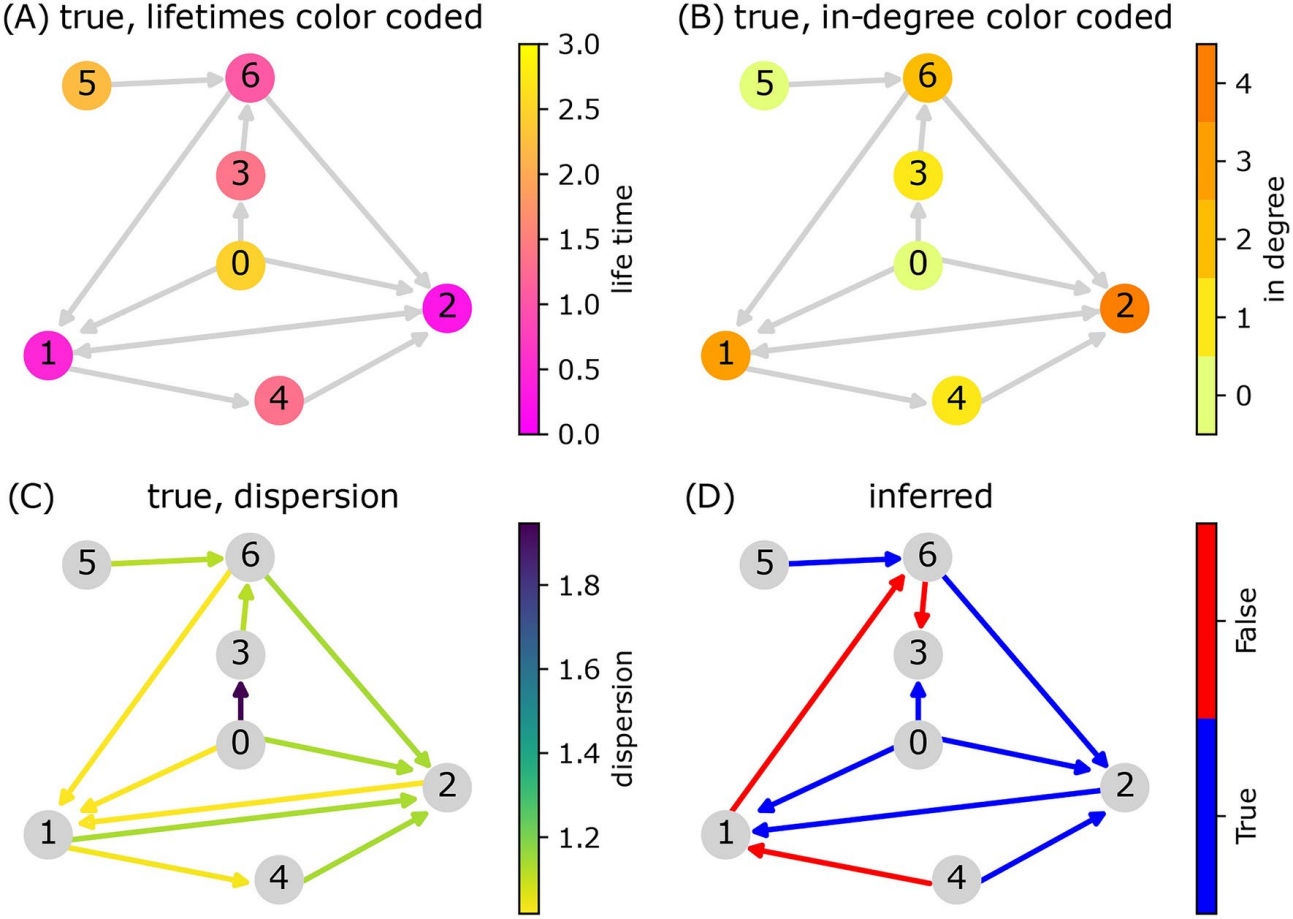
Simulation results for the *E. coli* gene regulatory network model. The features of the nodes and edges are color-coded according to the sub-figure titles and the provided color bars. Panels (**A**–**C**) display the true network, with each panel highlighting a specific feature in color: lifetimes and in-degree of the nodes in panels (**A**) and (**B**), and the index of dispersion which is associated with node-node correlation is shown in panel (**C**). Panel (**D**) presents the inference results, where correctly inferred directions are shown in blue, and incorrect ones are depicted in red.

Our strategy successfully infers the direction of the interaction between two nodes within an unknown network of interactions, where only the two-node joint steady-state distribution is given as the input, see Figs. 2, 3 and 4. It demonstrates that stationary probability distributions contain sufficient information to deduce the direction of a given interaction within an unknown network of interactions.

One of the most commonly used classifiers’ performance scores is the receiver operating characteristic curve (ROC)—which compares the true positive rate (TPR) to the false positive rate (FPR) at different threshold settings. A perfect classification yields TPR=1 with FPR=0, while a random one forms a diagonal line where FPR = TPR. The area under the receiver operating characteristic curve (AUC) is a single-valued score indicating overall performance, ranging from 0.5 for random to near 1 for perfect classification. We use these to evaluate the suggested direction inference strategy.

As aforementioned, our method poses a good direction classifier—it successfully infers the direction of a given edge where the relevant joint distribution is sufficiently sampled. As is shown in Fig. 2, the direction inference improves with a larger set of data points. In particular, a sufficiently large data set provides scores beyond the random classifier. It signifies that the direction of interaction is indeed encoded within the stationary snapshot of many data points. Importantly, recall that the input is simply the two-variables joint distribution, our method allows decomposition of the network. This means that within a given un-directed graph, one can learn the direction of a single edge independently, regardless of other edges, see further discussion in SI.

In the following, we discuss some key aspects that we found essential for the underlying goal. In particular, we examine some determinants that may affect probability to correctly infer directionality.

### Determinants and limitations

The stationary joint probability density between $x_i$ and all variables directly interacting with the *i*-th molecule must satisfy Eq. (1), hence it acts as a self-consistency test for stationarity. It is shown in ^48^ how this relation can be “inverted” to determine rate functions from observed probability densities. In other words, the information about the dynamical rate of a variable with its dependence on its coupled variables is indeed encoded within the joint steady-state distribution. However, while Eq. (1) must provably hold for all stationary states, empirically observed distributions carry sampling errors that can significantly impact inferred rates^48^, leading to potential errors in determining the interaction direction. Our results reflect this errors’ dependence, as illustrated in Fig. 2; larger *N*, which is equivalent to lower sampling errors, yields increased accuracy (SI).

A further characteristic examined is the index of dispersion *D*, i.e. the variance to mean ratio. It quantifies the variable dependence on others, where $D\approx 1$ indicates that the variable’s dynamic is weakly dependent on other variables (SI). We show in Fig. 3A that a higher probability of success inference is found where *D* is far from 1. We note that similar behavior was previously reported in ^48^. For a given edge $x_j \rightarrow x_i$ , the functional dependence of $r_i^+(x_j)$ in the level $x_j$ is better determined for higher mutual information between these two variables.

Moreover, some local properties of the network may affect the performance of our direction inference method. We examine the in- and out- degrees, and the lifetimes of the molecules. Recall that we evaluate the sensitivity of $\widetilde{r_i}(x_j)$ to $x_j$, where $\widetilde{r_i}(x_j)$ is a quantity averaged over all other income edges, thus we expect that the connectivity nature possesses an influence over the performance. Additionally, the lifetime of a molecule is also expected to play an important role in the overall performance as is discussed in ^48^. In the results presented in Fig. 3B we have found that the in-degree of $x_j$ presents some dependence over the performance, yet we did not find it significant, see further analysis and simulation results in SI. Moreover, we show in Fig. 3C that a lifetimes ratio far from 1 results in decreasing the probability for an accurately inferred direction of influence, which agrees with ^48^. Nevertheless, we comment that our network ensemble does not cover the entire possible network topologies space, an important factor that might skew the results, thus the nature of these phenomena needs further research.

### *E. coli* gene regulatory network

We aggregate the insights from the above, and apply our strategy for the direction inference on a previously proposed biologically realistic system—a model which is based on *E. coli* gene regulatory network provided in the DREAM challenge^52–54^, see “Methods”. The system, shown in Fig. 4 , poses various characteristic lifetimes and vertices’ degrees. We decompose the network and analyze each edge separately with its measured two-node joint distribution.

As shown, our inference method performs well for edges from nodes with zero in-degree with relatively long lifetimes (all edges from nodes 0 and 5), while erroneous direction emerges at edges connecting high-degree nodes with short lifetimes (nodes 1, 6 where each connected to two wrongly drawn arrows). We comment that our local two-node analysis may provide pathways that are not encoded in the true network, therefore complementary use of global analysis^56–61^ should be considered, especially when all other nodes were observed.

### Observation requirements

Our model is not restricted to a specific interpretation of the fluctuating variables (nodes). Generally, the set $\{x_i\}$ could represent levels of DNA, mRNA, complexes, proteins, or metabolites. For instance, our model applies to scenarios involving the expression levels of multiple genes (e.g., obtained via microarray or RNA-seq) alongside genotypes of cis-eQTL, similar to^62,63^. Another example is the two-dimensional data observations in^32^, where $x_i$ and $x_j$ are the measured mRNA and protein copy numbers. The authors used a yellow fluorescent protein fusion library to examine the single-cell profile of *E. coli*. Depending on the desired insights and measurable variables, one should select the most appropriate recording method.

As mentioned, we assume the process is governed by the stationary Master equation, with an interaction between the variables $x_i$ and $x_j$. The inference approach requires measuring $P(x_i, x_j)$ to determine the interaction orientation between these variables. Principally, the inference approach is quite general, allowing for varied underlying processes and biological interpretations of $x_i$ and $x_j$. For example, gene-regulatory networks can be modeled as discrete systems using the Master equation^49^. The Master equation can also describe spatial dependencies, such as complexes at a given codon or in modeling allosteric regulation. Furthermore, models can incorporate multiple gene transcription-translation processes, feedback loops, and ribosome stalling, to name only a few. These models may be simplified through “coarse-graining” or maintained in their complexity. The inference method thus requires the measurement of the joint probability $P(x_i; x_j)$ corresponding to the interpretation of each variable, see further discussion in the SI. Note that the interaction network is assumed to be static, unlike dynamic networks^14,62^.

## Discussion

Unveiling the interaction network among stochastic variables and delineating the direction of each interaction is crucial for understanding biological systems. Here, we present a method designed to infer the directionality within this network solely from the steady state of pertinent molecules. We assess the effectiveness of this approach across various properties of the system. Simulation results indicate that the proposed method demonstrates success in inferring the reaction direction in various cases.

As mentioned, our strategy relies on the time-invariant density, and as such possesses a benefit as explained in the following. Recorded data points can be employed without a known temporal order, taken at different sampling intervals, or aggregated from multiple experiments, as long as they were conducted under the same conditions. Hence this stationarity assumption enables the analysis of a wide range of biological systems. Importantly, Eq. (1) is not an approximation but corresponds to an exact flux balance relation at stationarity. The only dynamics excluded from our analysis is transient behavior such that stationary probability distributions are not accessible from experimental data. Even so, explicitly time-varying systems that technically never reach a stationary state, such as deterministic oscillations, may satisfy a similar form of Eq. (1) when considering their time-averaged probability distributions and rates. However, care has to be taken when comparing such time averages with population averages, see further discussion in SI.

The static snapshot provides statistical features of the stochastic variables, enabling the inference of the interaction direction. Statistical information is frequently used to gain insights from these snapshots about the dynamics and the relations between variable components^29,38,39,64–66^. Our strategy for revealing interaction directions thus constitutes a previously unknown intermediate approach, placed between purely statistical methods used to infer effective connectivity (such as correlations) and approaches that infer physical connectivity from high-resolution, time-ordered recordings of the complete dynamics^26,27,34,67,68^. In particular, statistical methods for inferring *directed* edges that rely on partial correlation analyses were applied for revealing directed graphs^42,44,69,70^. These methods, however, require measurements from other components within the system and in that sense are non-local. Nevertheless, we tested the performance of the partial correlation analyses over our synthetic data and found that it is underachieved and thus inadequate for our goal (see SI).

Using the sensitivity analysis presents an equivalence with the examination of the slope of ${\widetilde{r}}_{ik}(x_j)$ or its linear response. This linear sensitivity analysis for the interaction network identification is conceptualized and demonstrated for stationary systems under small perturbations^29^. There, the authors assume continuum variables that evolve via coupled differential equations with no noise or with additive noise. Conversely, our method considers discrete variables that propagate following the Master equation which effectively possess multiplicative noise. In addition, we note that our approach does not require externally driving the system in a controlled way that is not applicable in many systems^29^.

Another possible directionality classification quantity is related to the Sum-of-Squared Errors (SSE), which might be associated with the Minimum Description Length (MDL). The SSE (or the MDL) comparison between the two directions is well mathematically established^71–73^. Nevertheless, we show (see SI) that our directionality classification quantity based on $|J_{ij}|$ outperforms the previously proposed SSE-based one, especially in low *N*. In the SI we further discuss the classifying quantities, with additional simulation results.

Harnessing of the global flux balance Eq. (1) to gain insight on the interaction quantification has been suggested recently^48^. There, the authors demonstrated that the steady state joint distribution indeed encapsulates information about the dynamical features such as the birth rates. They assume that the direction of the edge, together with the stationary $P(x_i,x_j)$, are given, and thus find the functional shape of $r_{i}^+(x_j)$ for various values of $x_j$. Here we exploit this approach, but instead of looking for the full functional shape we only examine a partial behavior of $r_{i}^+(x_j)$ - its sensitivity analysis - without assuming the direction of the edge at all, see further discussion on SI.

Furthermore, we have examined some of the performance determinants, i.e. the properties which affect the efficacy of the direction’s identifier. For example, we examined the variables’ degradation rate. When upstream and downstream variables fluctuate with similar lifetimes, we find a greater probability of successfully inferring their edge direction. Furthermore, we have found a connection between the index of dispersion *D* and the ability to infer the direction of the interaction, where larger *D* yields a larger success ratio. Both degradation times and index of dispersion effect on the overall performance were previously demonstrated to be with similar conclusions as is given in^48^. Moreover, some of the network topological features such as the node degree were also being inspected. We have found that a lower incoming degree of the node provides better results in the inference of the direction. That is in agreement with previously published results that show that increasing the incoming degree requires more data points to gain the same quality of inference^29,65^.

To summarize, the method performs well where the joint distribution is well sampled. That is achieved when measuring sufficiently many data points such that they satisfactory capture the required statistical features. Nevertheless, as mentioned, here we provide only a basic algorithm that proves the principle that the direction of interaction is encoded within the stationary information.

For future research, we suggest examining more sophisticated approaches, such as deep learning methods. Previously published techniques involve artificial neural networks to study node-to-node interactions^74–76^, while others employ stacking methods^77–79^. Supervised learning methods, such as regression-based approaches, are commonly used to infer the global structure of gene regulatory networks^56–61^. These methods, however, require multidimensional data that goes beyond simple two-node observations. It is important to note that for these learning methods, the training data must closely resemble the observational data under scrutiny, a condition that can be challenging to fulfill in real-world situations. Moreover, these approaches typically require very large datasets and significant training resources, including time and GPUs, limitations that might be eased in the future with advances in technology. Yet, as noted in^55^, there is currently no universal method that applies to all scenarios; there is no “one-size-fits-all” solution, and the inference method should be tailored to the system under investigation.

## Methods

### Background theory

The well-known Master equation for *V* variables yields at stationarity the global balance relation, which means that for every variable *i*

2 $$\begin{aligned} 0 = \sum _k \langle r_{ik}(\vec {x} - \vec {d}_{ik})|x_i -d_{ik}\rangle { P}(x_i -d_{ik}) - \langle r_{ik}(\vec {x})|x_i \rangle { P}(x_i ). \end{aligned}$$

$\vec {d}_{ik}=\{0, \dots , d_{ik}, \dots , 0\}$ where the non-zero value is located in the *i*-th component. Each $x_i$ denotes the quantity of units of type *i*. For instance, this could represent the mRNA or protein copy number indicative of gene expression levels, a metabolite level within a cell, or the population count of a given species.

Here, we emphasize that the system contains *V* variables, so the state of a particular sample is given by the *V*-dimensional array $\{x_1, x_2, \cdots , x_V\}$. Assuming the system is sampled *N* times, the observed dataset contains $V \times N$ points in total.

The above relation is derived from a simple summation of the stationary Master equation over all other variables, and has been derived and discussed previously^45–48^, see also derivation in SI. The angular brackets represent conditional means, i.e. $\langle r_{ik}(\vec {x})|x_i\rangle \equiv \sum _{{x_j}, j\ne i} r_{ik}(\vec {x}) P(x_1, x_2, \dots x_{i-1}, x_{i+1}, \dots x_N | x_i )$^80^. Equation (2) can be wriiten as

3 $$\begin{aligned}  \sum _k \langle {\widetilde{r}}_{ik}(x_i -d_{ik}, x_j)|x_i -d_{ik}\rangle { P}(x_i -d_{ik}) = \quad \langle {\widetilde{r}}_{ik}(x_i, x_j)|x_i \rangle { P}(x_i ) \end{aligned}$$

where ${\widetilde{r}}_{ik}(x_i, x_j) \equiv \langle r_{ik} (\vec {x}) |x_i, x_j \rangle .$ Our approach is based on *local* sensitivity of ${\widetilde{r}}_{ik}$ to $x_j$, which aims to quantify the dependence of the dynamic of $x_i$ on the level of $x_j$. In particular, we calculate $J_{ij}\equiv \partial r_{ik} / \partial x_j$ next to the probable abundance point. The quantities of $|J_{ij}|$ and $|J_{ji}|$ are then utilized as a classification feature. The nature of this comparison between $|J_{ij}|$ and $|J_{ji}|$ , and its role in classification would depend on the specific context or system. It is partially inspired by^71,72^ where both directions were quantified using regression and then assessed such that the more probable direction is chosen.

### Models

For numerical demonstrations, we generate a random directed graph ${\hat{G}}$ which holds the topology of the network. Mathematically the graph is described by the adjacency matrix with elements $G_{ij}=1$ where the state of *j* affects the dynamics of node *i*, i.e. $j \rightarrow i$, and $G_{ij}=0$ otherwise. We examined systems with Erdös Rényi random networks. In addition, we examine more biologically realist system which is based on a subset of an *E. coli * gene regulatory network and was provided in the DREAM challenge^52–54^.

For illustration, we use a typical reaction rate model where a molecule reaction can be either a reproduction $d_i^+ =+1$ or degradation $d_i^- =-1$. We specified that the production rate of $x_i$, denoted as $r_i^+$, depends on other units’ states via the reaction network and that each molecule within the system degrades independently with a typical degradation rate. Hence, Eq. (2) can be written as

4 $$\begin{aligned} \langle {r}_{i}^+(\vec {x})|x_i\rangle { P}(x_i) = \frac{1}{\tau _i}(x_i+1)P(x_i+1) \end{aligned}$$

where $r_i^+(\vec {x})$ encodes all incoming edges (all units that affect the production rate of node *i*), and $\tau _i$ is the known lifetime of molecule *i*.

#### Michaelis–Menten regulatory network

The birth rate of molecule *i* is

5 $$\begin{aligned} r_i ^+ = \sum _{j\ne i}G_{ij}\frac{\lambda x_j}{x_j+k} + 30 \cdot \delta (\sum _{j\ne i}G_{ij}) \end{aligned}$$

where for our simulation we choose $V=10$ nodes, 10 directed edges, $\lambda =100$ and $k=100$. These commonly described production rates in biochemical reaction networks, where $\lambda$ gives the maximal rate and *k* refers to the concentration which gives half of the maximal rate^28,81^. The degradation rates follow $r_i^-=x_i$ for all *i*.

We note that the Michaelis–Menten model describes the dynamics that involve enzymes. This rate function holds for a quasi-steady state with low enzyme concentrations^28,81^. In the context of gene regulation through transcription factor (TF) binding to gene regulatory sequences, the application of a Michaelis–Menten rate type is considered valid due to the typical excess TFs over their chromosome-binding sites^82^. Still, when intermediate molecules are involved, or when describing dynamics of comparable concentrations, the Michaelis–Menten-rate law needs alternatives^83^.

#### Coupled Goodwin oscillators

Each Goodwin oscillator is given by three interacting variables $(x_i,y_i,z_i)$ which are coupled through the *y* variables. Their proliferation rates are given in the following:

6 $$\begin{aligned} r_{x,i} ^+= & \frac{\lambda _{x}}{(z_i/k)^n+1} \nonumber \\ r_{y,i} ^+= & \lambda _{y} x_i + \sum _{j\ne i}G_{ij} y_j \nonumber \\ r_{z,i} ^+= & \lambda _{z} y_i \end{aligned}$$

and death rates are $r_i^-=x_i$ for all *i*. Here, the dynamic influences network within each oscillator is known, namely the relations within the triplet $(x_i, y_i, z_i)$ are given, hence we aim to infer only the coupling network between these triplets $G_{ij}$. In the simulation presented in the main text, ${\hat{G}}$ has 8 edges and 8 nodes—where each node is a triplet, such as we have 24 fluctuated variables in total.

#### Gene regulatory interactions in *E. coli*

As mentioned, we also examine a more biologically realist model which is based on a subset of an *E. coli * gene regulatory network and was provided in the DREAM challenge^52–55^. There, the *in-silico* network inference challenge investigated how well gene networks can be deduced from simulated data. The network is derived as subgraphs from the recognized *E. coli* and *S. cerevisiae* gene regulation networks^84^. This means that the results presented are thus biologically realistic, i.e. aiming to capture a reasonable network, but not given from a real observation, and the gene indexes are thus arbitrary.

There, the variables $x_i$ are described as continuous and possess small values. Here, for the discrete treatment of the model, we scaled the variables by ${\tilde{x}}_i=x_i/100$, thus our *E. coli * gene regulatory network is thus defined and simulated with the following rates:

7 $$\begin{aligned} r_1^+= & \frac{ k_{0,1}}{K_{M,1}+D} \nonumber \\ r_2^+= & \frac{ k_{0,2} }{K_{M,2}+{\tilde{x}}_1} + \frac{ k_{0,3} {\tilde{x}}_3 }{K_{M,3}+{\tilde{x}}_3} + \frac{ k_{0,4} {\tilde{x}}_7 }{K_{M,4}+{\tilde{x}}_7} \nonumber \\ r_3^+= & \frac{ k_{0,5} {\tilde{x}}_1}{K_{M,5}+{\tilde{x}}_1} + \frac{ k_{0,6}}{K_{M,6}+{\tilde{x}}_2} + \frac{ k_{0,7} {\tilde{x}}_5 }{K_{M,7}+{\tilde{x}}_5} + \frac{ k_{0,8} {\tilde{x}}_7}{K_{M,8}+{\tilde{x}}_7} \nonumber \\ r_4^+= & \frac{ k_{0,9}}{K_{M,9}+{\tilde{x}}_1} \nonumber \\ r_5^+= & \frac{ k_{0,10}}{K_{M,10}+{\tilde{x}}_2} \nonumber \\ r_6^+= & \frac{ k_{0,11} U}{K_{M,11}+U} \nonumber \\ r_7^+= & \frac{ k_{0,12} {\tilde{x}}_4}{K_{M,12}+{\tilde{x}}_4} + \frac{ k_{0,13} {\tilde{x}}_6}{K_{M,13}+{\tilde{x}}_6} \end{aligned}$$

and for every *i*

8 $$\begin{aligned} r_i^- = \gamma _i {\tilde{x}}_i. \end{aligned}$$

with parameters

**Table:** 

Gene	Parameter values
$N_1$	$k_{0,1} = 0.0362$, $K_{M,1} = 0.1259$, $\gamma _1 = 0.4060$,
$N_2$	$k_{0,2} = 1.0106$, $K_{M,2} = 1.7937$, $k_{0,3} = 0.3550$,
	$K_{M,3} = 1.2069$, $k_{0,4} = 0.7472$, $K_{M,4} = 1.2858$,
	$$\gamma _2 = 2.1362$$
$N_3$	$k_{0,5} = 2.4007$, $K_{M,5} = 0.8218$, $k_{0,6} = 0.8511$,
	$K_{M,6} = 1.7099$, $k_{0,7} = 2.8247$, $K_{M,7} = 1.6656$,
	$k_{0,8} = 0.6081$, $K_{M,8} = 0.0202$, $\gamma _3 = 3.8740$,
$N_4$	$k_{0,9} = 0.0903$, $K_{M,9} = 0.069$, $\gamma _4 = 0.7256$
$N_5$	$k_{0,10} = 0.5264$, $K_{M,10} = 0.9600$, $\gamma _5 = 0.7466$
$N_6$	$k_{0,11} = 0.6541$, $K_{M,11} = 1.0891$, $\gamma _6 = 0.4525$
$N_7$	$k_{0,12} = 0.0090$, $K_{M,12} = 0.5191$, $k_{0,13} = 1.1236$,
	$K_{M,13} = 0.4986$, $\gamma _{7} = 0.9473$

These parameters are taken from^52–54^. The ‘external disturbance’ is modeled by uniform distribution, means $D, U \sim \textrm{Uniform}[0,1]$.

## Supplementary Information

Supplementary Information.

## Data Availability

All data are included in the main article and its supplementary information files.
