# Supplementary material for: Determining interaction directionality in complex biochemical networks from stationary measurements
Source: Sci Rep. 2025 Jan 23;15:3004. doi: 10.1038/s41598-025-86332-0 (PMC11758029; doi:10.1038/s41598-025-86332-0)
Supplement: Supplementary file 1 — Supplementary Information. [file 41598_2025_86332_MOESM1_ESM.pdf]

SUPPLEMENTARY INFORMATION

Appendix A: Mathematical Background and Derivations

We consider a system with V variables which pose the networks' nodes. Consider the multi-dimensional stationary master equation

$$0 = \sum_k \sum_{i=1}^{V} \left[r_{ik}(\vec{x} - \vec{d}_{ik}) P(\vec{x} - \vec{d}_{ik}) - r_{ik}(\vec{x}) P(\vec{x}) \right]. \tag{A1}$$

r_{ik} represents the k-th reaction rate of molecule i and $\vec{d}_{ik} = \{0, \dots, d_{ik}, \dots, 0\}$ where the non-zero value is located in the i-th component. In the next step we perform a summation over the support of $P(\vec{x})$ for (V-1) variables, excluding one variable from the summation. Without loss of generality, we exclude from summation the variable x_1, meaning that for every $i > 1$ we sum over all the possibilities of x_i, and find

$$0 = \sum_{x_2=0}^{\infty} \cdots \sum_{x_V=0}^{\infty} \left\{ \sum_k \sum_{i=1}^{V} \left[r_{ik}(\vec{x} - \vec{d}_{ik}) P(\vec{x} - \vec{d}_{ik}) - r_{ik}(\vec{x}) P(\vec{x}) \right] \right\} \tag{A2}$$

Following the conditional probability relation $P(x_1, x_2 \dots x_V)/P(x_1) = P(x_2 \dots x_V | x_1)$ [1], we obtain $\sum_{x_2=0}^{\infty} \cdots \sum_{x_V=0}^{\infty} f(\vec{x}) P(\vec{x}) = \langle f(\vec{x}) | x_1 \rangle P(x_1)$ for an arbitrary function $f(\vec{x})$. Therefore, we can write

$$0 = \sum_k \left[\langle r_{1k}(\vec{x} - \vec{d}_{1k}) | x_1 \rangle P(x_1 - d_{1k}) - \langle r_{1k}(\vec{x}) | x_1 \rangle P(x_1) \right] \tag{A3}$$

which recovers Eq. (1) in the main text. This relation though depends on the levels of all variables within the system via $P(x_1, x_2, \dots x_V)$.

In the case presented in the main text, $d_{1k} = \pm 1$, then

$$\langle r_1^+(\vec{x}) | x_1 \rangle P(x_1) = \langle r_1^-(\vec{x} + \vec{e}_1) | x_1 + 1 \rangle P(x_1 + 1). \tag{A4}$$

We assume that the birth rate depends on the other molecules levels, and only self-degradation apply. Then

$$\begin{aligned} \langle r_1^+(\vec{x}) | x_1 \rangle P(x_1) &= \sum_{x_2=0}^{\infty} \sum_{x_3=0}^{\infty} \cdots \sum_{x_V=0}^{\infty} r_i^+(x_2, x_3, \dots x_n) P(x_2, x_3, \dots x_V | x_1) P(x_1) \\ \langle r_1^-(\vec{x} + \vec{e}_1) | x_1 + 1 \rangle P(x_1 + 1) &= \beta_1 (x_1 + 1) P(x_1 + 1), \end{aligned} \tag{A5}$$

where $\beta_1 \equiv \tau_i^{-1}$ represents the self-degradation rate of type 1. Thus, back to generality we re-index $1 \to i$, and obtain

$$\langle r_i^+(\vec{x}) | x_i \rangle P(x_i) = \beta_i (x_i + 1) P(x_i + 1), \tag{A6}$$

where similarly β_i represents the self-degradation rate of type i. The latter can be written as

$$\begin{aligned} \langle r_i^+(\vec{x}) | x_i \rangle P(x_i) &= \sum_{x_j=0}^{\infty} \langle r_i^+(\vec{x}) | x_j \rangle P(x_j | x_i) P(x_i) \equiv \sum_{x_j=0}^{\infty} \widetilde{r}_i^+(x_j) P(x_j | x_i) P(x_i) \\ &= \langle \widetilde{r}_i^+(x_j) | x_i \rangle P(x_i) = \beta_i (x_i + 1) P(x_i + 1). \end{aligned} \tag{A7}$$

From the above relation one can determine $\widetilde{r_i}^+(x_j)$ as a function of x_j from stationary distribution $P(x_i, x_j)$ solely as shown in [2].

Appendix B: A Comment About Non-Stationary Processes

Consider the general time-dependent Master equation

$$\partial_t P_n(t) = \sum_{k \neq n} a_{kn} P_k(t) - \sum_{k \neq n} a_{nk} P_n(t). \tag{B1}$$

Applying time averaging on both sides yields

$$\frac{P_n(T)-P_n(0)}{T} = \sum_{k\neq n} a_{kn}\frac{1}{T}\int_0^T P_k(t) - \sum_{k\neq n} a_{nk}\frac{1}{T}\int_0^T P_n(t) \tag{B2}$$

$$\frac{P_n(T)-P_n(0)}{T} = \sum_{k\neq n} a_{kn}\overline{P_k}(T) - \sum_{k\neq n} a_{nk}\overline{P_n}(T), \tag{B3}$$

which presents a similar form to stationary Master equation Eq. (A1) in cases where $\lim_{T\to\infty}\frac{P_n(T)-P_n(0)}{T}=0$ and the probability distributions are replaced with their time averaged ones.

Appendix C: Simulations Details

After presenting our mathematical framework, we examine how useful is our method to analyze noisy data from finite measurements. The time propagation simulations, that had been done using the Gillespie algorithm, are for the purpose of mimicking various experimental scenarios.

From the stochastic processes described in Methods, we measure the stationary probability density function (PDF) $P(x_i,x_j)$ as explained in the following. We first construct a long time realization of the variables under consideration. Such a realization is routinely constructed using the Gillespie algorithm [3]. In our simulation, we use $2\cdot 10^7\cdot V$ time steps, where V is the number of variables simulated. We assume that the distribution obtained from that very-long realization is thus exact. Then, we sample from the "exact" PDF a given number of data points N corresponding to their probability.

Here we use the same algorithm used in [2] to infer $\widetilde{r}_i^+(x_j)$ from $P(x_i,x_j)$. There, the authors found the birth rate $\widetilde{r}_i^+(x_j)\equiv\vec{f}$ by finding

$$\underset{f}{\operatorname{argmin}}\left\{\left|\hat{G}\vec{f}-\vec{h}\right|^2+\epsilon\left|\hat{\Gamma}\vec{f}\right|^2\right\} \qquad \text{subject to } \vec{f}\geq 0 \tag{C1}$$

where we defined $G_{mn}\equiv P(x_i=m,x_j=n)$, $f_n\equiv\widetilde{r_i}^+(x_j=n)$ and $h_m\equiv\beta_i(m+1)P(x_i=m)$. The additional regularization matrix $\Gamma_{m,n}\equiv\delta_{m,n}-\delta_{m,n+1}+\delta_{m,n+2}$ penalizes non-smooth functions with regularization constant $\epsilon=N^{-1/2}$ where N is the number of sampled data points, see [2].

Appendix D: The Distribution of the Conditional Expectation

Aforementioned, Eq. (1) in the main text is precise, with no approximations made during its derivation, see App. A. Nonetheless, a finite number of sampling points, represented by N, introduces errors in $P(x_i)$, which in turn affects the estimated $\widetilde{r_i}^+(x_j)$, potentially leading to erroneous conclusions about the direction of interaction.

Here, we use examples where the proliferation rate may depend on other molecules' level $r_i^+(\vec{x})$, and the death rates are self govern degradation $r_i^-(x_i)=\beta_i x_i$ similarly as introduced in App. A. hence

$$\langle r_i^+(\vec{x})|x_i\rangle = \frac{\beta_i(x_i+1)P(x_i+1)}{P(x_i)}. \tag{D1}$$

We note that the *measured* $P(x_i)$, marked as $\hat{P}(x_i)$, is essentially a random number where noise is induced by the finite sampling. The probability that from N samples, we find k realizations with level x_i of molecules from type i is given by

$$\mathrm{PDF}(k) = \binom{N}{k} P(x_i)^k(1-P(x_i))^{N-k}. \tag{D2}$$

Then, by a simple transformation, we obtain that the estimated (measured) value $\hat{P}(x_i)$ is distributed via

$$\mathrm{PDF}\left[\hat{P}(x_i)=\frac{k}{N}\right] = \frac{P(x_i)^{N\hat{P}(x_i)}\left[1-P(x_i)\right]^{N-N\hat{P}(x_i)}}{B\left[N\hat{P}(x_i)+1,N-N\hat{P}(x_i)+1\right]}, \tag{D3}$$

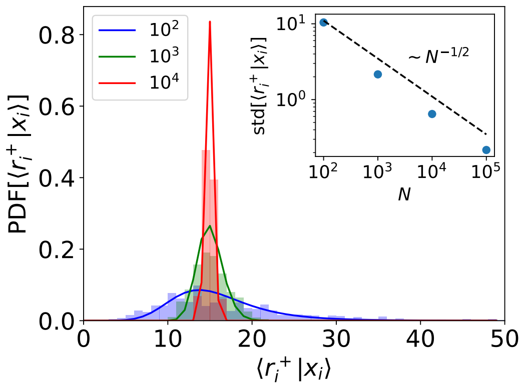

FIG. 1. The distribution PDF $\left[z = \langle r_i^+(\vec{x})|x_i\rangle\right]$ evaluated at $x_i = \text{ArgMax}[P(x_i)]$. It is approximated as correlated Gaussian ratio distribution, and evaluated using Geary-Hinkley transformation Eq. (D5). Inset: The measured standard deviation for large N falls as $N^{-1/2}$.

where $B[\alpha, \beta]$ refers to the Beta function. When $N \gg 1$, $NP(x_i) \gg 1$ and $N[1 - P(x_i)] \gg 1$ we find

$$\hat{P}(x_i) \sim \mathcal{N}\left[P(x_i), \frac{P(x_i)\,(1 - P(x_i))}{N}\right] \tag{D4}$$

which is the Gaussian distribution with mean $\langle \hat{P}(x_i)\rangle = P(x_i)$ and variance $\text{Var}\left[P(\hat{x_i})\right] = P(x_i)\,[1 - P(x_i)]\,/N$.

Now, the distribution of the evaluated $z = \langle r_i^+(\vec{x})|x_i\rangle$ is controlled by the ratio of two random numbers. In the case where both the numerator and the denominator are Gaussian distributed, we can use the transformation

$$t \approx \frac{\mu_1 z - \mu_2}{\sqrt{\sigma_1^2 z^2 - 2\rho\sigma_2\sigma_1 z + \sigma_2^2}}. \tag{D5}$$

with $\mu_1 \equiv \langle \hat{P}(x_1)\rangle$, $\mu_2 = \langle \beta_i(x_i+1)\hat{P}(x_i+1)\rangle$,$\sigma_1^2 \equiv \text{Var}\left[\hat{P}(x_i)\right]$, $\sigma_2^2 \equiv \text{Var}\left[\beta_i(x_i+1)\hat{P}(x_i+1)\right]$ and ρ is the Pearson correlation coefficient between the numerator and the denominator. Then $t \sim \mathcal{N}(0,1)$ following the Geary-Hinkley theorem [4, 5]. Note that PDF $\left[z = \langle r_i^+(\vec{x})|x_i\rangle\right]$ is a wide distribution, yet simulation results suggest that when the distributions of the numerator and denominator are sufficiently narrow, the standard deviation of z decreases as $1/\sqrt{N}$, where N is the number of samples. This is true only where $\hat{P}(x)$ is Gaussian distributed, i.e. where x is in the central part of $P(x)$. In Fig. 1 we illustrate the statements given above.

Appendix E: Dependence of the number of subdivisions and batch size of the data

Consider that the total data length is N, which may represent the total number of samples or the data points. Then we divide this set into S equal-sized sets, namely batches, where the size of each batch is b (note that $N = S \cdot b$). With the same amount of data points N, one may find different quality of estimation depending on the number of the subdivisions S and the batch size b. We have found by simulation that for a given N, there is an advantage of taking small batches with $b = 10$ samples, see Fig. 2 . This phenomenon might be partially explained by the following. Too low batch size b might be associated with underfitting - where the amount of information within each batch is insufficient to make any inference. Yet, there is an essential non-linear relation between the sampling error of $P(x_i, x_j)$ and the quality of estimation (see previous section).

Appendix F: Index of Dispersion

In cases where x_i is independent on other variables; means independent proliferation $r_i^+ = \text{const.} = c_i$ with degradation $r_i^- = \beta_i x_i$, we find $P(x_i) = e^{-c_i/b_i}(c_i/b_i)^n/x_i!$. The latter is a Poisson distribution with index of dispersion $\text{var}(x_i)/\langle x_i\rangle \equiv D = 1$.

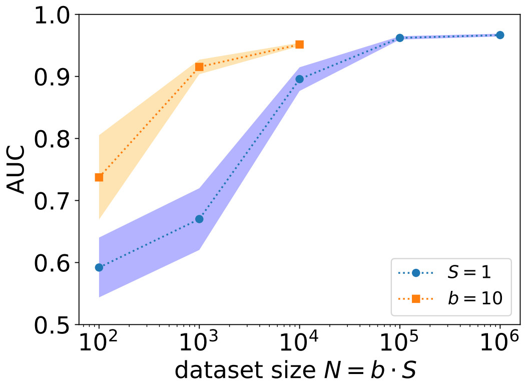

FIG. 2. Using small batches might be beneficial, especially for small N. The results are given from the Goodwin oscillators model.

The stationary Master equation where $r_i^+(\vec{x})$ may generally depends on other molecules' levels and degradation $r_i^-(x_i) = \beta_i x_i$ is

$$0 = \sum_{i=1}^{V} \left[r_i^+(\vec{x} - \vec{e}_i) P(\vec{x} - \vec{e}_i) - r_i^+(\vec{x}) P(\vec{x}) + \beta_i (x_i + 1) P(\vec{x} + \vec{e}_i) - \beta_i x_i P(\vec{x}) \right] \tag{F1}$$

where we simply used Eq. (A1) with two possible reactions for each molecules $d_{ik} = d_i^{\pm} = \pm 1$. Multiply by x_1 and sum over all possible states $\sum_{x_1=0}^{\infty} \cdots \sum_{x_V=0}^{\infty}$ yields

$$0 = \sum_{x_1=0}^{\infty} \cdots \sum_{x_V=0}^{\infty} \sum_{i=1}^{V} x_1 \left[\underbrace{r_i^+(\vec{x} - \vec{e}_i) P(\vec{x} - \vec{e}_i) - r_i^+(\vec{x}) P(\vec{x})}_{\mathrm{I}} + \underbrace{\beta_i (x_i + 1) P(\vec{x} + \vec{e}_i) - \beta_i x_i P(\vec{x})}_{\mathrm{II}} \right]. \tag{F2}$$

Then

$$\sum_{x_1=0}^{\infty} \cdots \sum_{x_V=0}^{\infty} \sum_{i=1}^{V} x_1 \cdot (\mathrm{I}) = \langle r_1^+(\vec{x}) \rangle \tag{F3}$$

$$\sum_{x_1=0}^{\infty} \cdots \sum_{x_V=0}^{\infty} \sum_{i=1}^{V} x_1 \cdot (\mathrm{II}) = \sum_{x_1=0}^{\infty} \cdots \sum_{x_V=0}^{\infty} \sum_{i=1}^{V} x_1 \beta_i (x_i + 1) P(\vec{x} + \vec{e}_i) - x_1 \beta_i x_i P(\vec{x}) = \beta_1 \langle x_1 \rangle$$

Thus we find that $\beta_1 \langle x_1 \rangle = \langle r_1^+(\vec{x}) \rangle$. Similarly, we multiply by x_1^2 and find

$$\sum_{x_1=0}^{\infty} \cdots \sum_{x_V=0}^{\infty} \sum_{i=1}^{V} x_1^2 \cdot (\mathrm{I}) = 2 \langle x_1 r_1^+(\vec{x}) \rangle + \langle r_1^+(\vec{x}) \rangle \tag{F4}$$

$$\sum_{x_1=0}^{\infty} \cdots \sum_{x_V=0}^{\infty} \sum_{i=1}^{V} x_1^2 \cdot (\mathrm{II}) = \beta_1 \langle x_1 \rangle - 2 \beta_1 \langle x_1^2 \rangle$$

Using the above we find

$$\begin{aligned} 2\beta_1 \langle x_1^2 \rangle &= \beta_1 \langle x_1 \rangle + 2 \langle x_1 r_1^+(\vec{x}) \rangle + \langle r_1^+(\vec{x}) \rangle \\ 2\beta_1 \langle x_1^2 \rangle - 2\beta_1 \langle x_1 \rangle^2 &= \beta_1 \langle x_1 \rangle + 2 \langle x_1 r_1^+(\vec{x}) \rangle + \langle r_1^+(\vec{x}) \rangle - 2 \langle r_i^+(\vec{x}) \rangle \langle x_1 \rangle \\ \beta_1 \mathrm{var}(x_1) &= \beta_1 \langle x_1 \rangle + \mathrm{cov}\left(x_1, r_i^+(\vec{x})\right) \end{aligned} \tag{F5}$$

Hence we obtain

$$D \equiv \frac{\mathrm{var}(x_1)}{\langle x_1 \rangle} = 1 + \frac{\mathrm{cov}\left(x_1, r_1^+(\vec{x})\right)}{\langle r_1^+(\vec{x}) \rangle}. \tag{F6}$$

Where the index of dispersion D, also called the Fano factor, effectively quantifies the contribution of the correlation between $r_i^+(x_j)$ and x_i, to the variability of x_i as was previously shown in [6]. In cases where x_i is weakly depend on other variables; means $r_i^+ \approx$ const. we we find $D \approx 1$.

Appendix G: Network Topological Properties

We survey several topological properties of the network and their effect on the overall performance. In particular, we examine the local topological properties for the edge $x_i \to x_j$, which includes the in- and out-degree of both x_i and x_j, see Fig. 3. Our simulations do not significantly indicate the influence of local degrees on the performance, yet a slight dependence observed in the in-degree of x_i as shown in the main text.

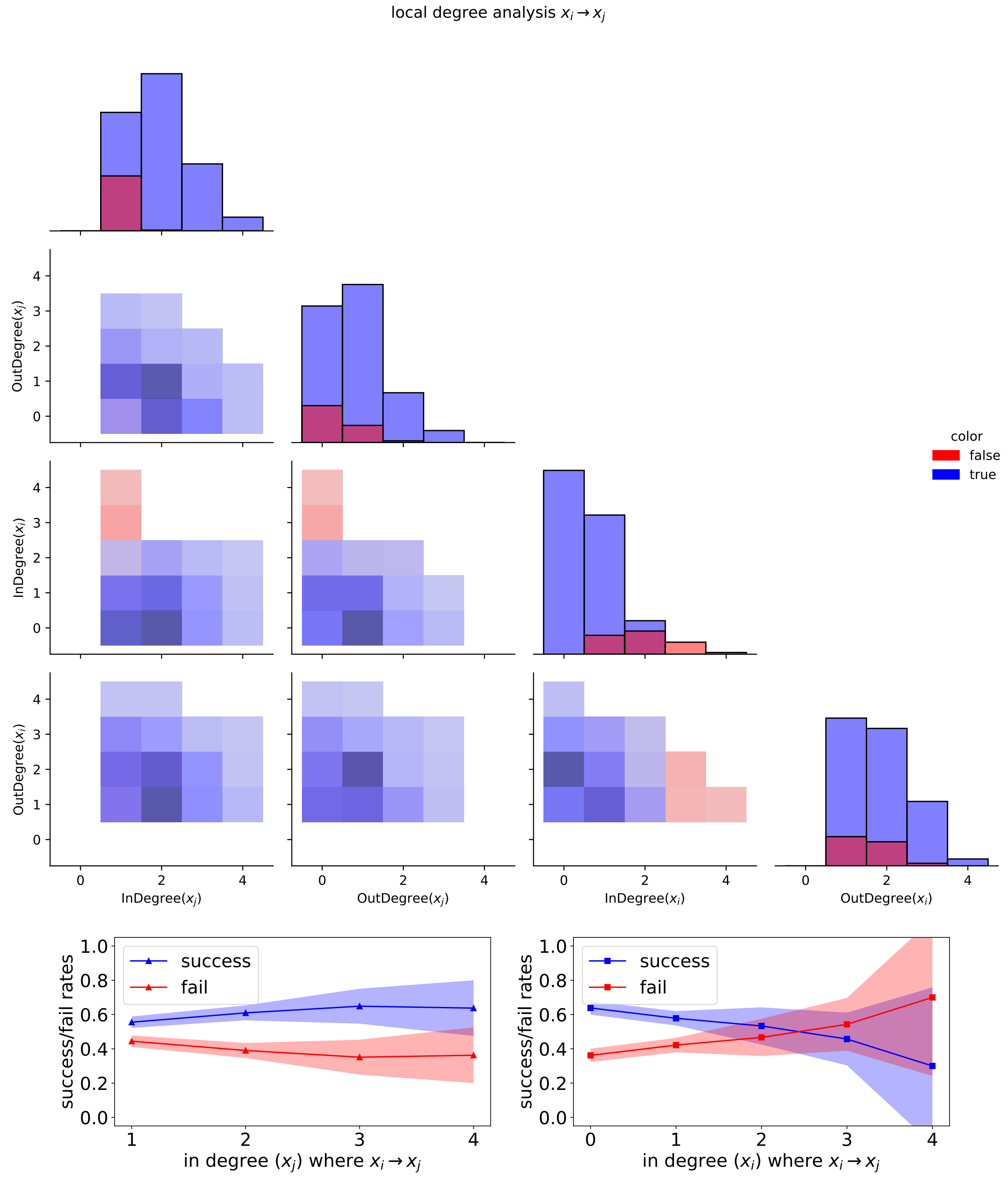

FIG. 3. local degree analysis on the overall performance. Upper panel presents results between pairs of features (=degrees). In the lower row we show the results for the in-degree of x_i and x_j (the former is the same as in the main text).

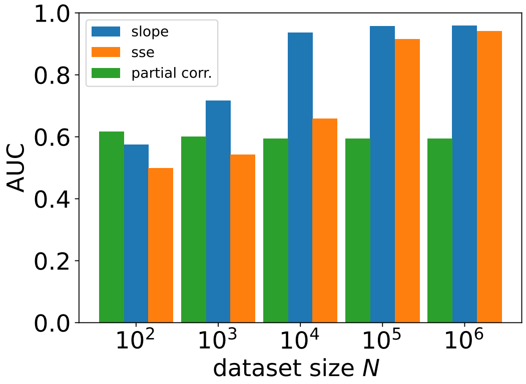

FIG. 4. Examination of classification features. We found that the classifying quantity which is the slope at some $\vec{x}^*$, namely $|J_{ij}|$, out-performs the SSE-based feature especially in low N. In addition, we have recorded all other variables within the network to evaluate the partial correlation.

Appendix H: Classification Features

1. Considered Attributes

We have tested three attributes, i.e. quantities, which aim to capture the strength of association from one variable to the other. The first one is based on the sensitivity analyses of the dynamic rate. There the attribute is the slope $|J_{ij}|$ - which quantifies the changes in the rate of x_i, i.e. $\tilde{r}_i(x_j)$, while changing the level of another variable x_j. That attribute was used throughout the main text and the SI up to here.

Another classification attribute that was examined is described in the following. In [7, 8] the authors suggested using the Minimum Description Length (MDL) principle which is described as a proxy for the Kolmogorov complexity. There they define a quantity $\Delta_{x\to y} = [L(x) + L(y|x)] / [L(x) + L(y)]$ and $\Delta_{y\to x}$ is defined analogously. Then infer $x \to y$, if $\Delta_{x\to y} < \Delta_{y\to x}$ holds up to an additive constant. The MDL is approximately the sum-of-squared errors (SSE). Here we quantify the SSE where assuming that $\tilde{r}_i(x_j)$ indeed depends on x_j, the SSE where assuming $\tilde{r}_i$ is independent of x_j, as well as the SSE of $\tilde{r}_j(x_i)$ and $\tilde{r}_j$. The so-called SSE attribute presented in Fig. 4

The last classification attribute we examine is the partial correlation, which disadvantageously requires recording all variables within the system. We have found that the partial correlation feature exceeds the completely random classifier, i.e. it exceeds AUC=0.5. However, it does not necessarily yield better results and thus is not preferred over the other classification features, particularly for relatively high N. The results are presented in Fig. 4 with an agreement with [9].

2. Feature Engineering - Oriented Graphs

Hitherto, we consider the undirected edge (i, j) is given with its corresponding stationary joint distribution $P(x_i, x_j)$ and aim to determine its binary direction, which means either $i \to j$ or $j \to i$. In graph theory that task is called 'graph orientation', which aims to assign a single direction to every edge in an undirected graph.

Let consider the attribute $J_{ij} \equiv \frac{d\tilde{r}_i(x_j)}{dx_j}|_{x_j=x_j^*}$. Thus, for a given edge we infer its direction as follows; if $|J_{ij}| > |J_{ji}|$ an arrow from j to i is drawn, and vise-versa - if $|J_{ij}| \geq |J_{ji}|$ we infer an arrow from i to j. It gives that the classification feature is in effect the quantity $|J_{ij}| - |J_{ji}|$. Throughout the main text and the SI up to here, the results are presented for the binary classification, with a preprocess of the features engineering as described above.

Appendix I: Further Discussion on the Models

The suggested inference approach presented in the main text uses a well-established mathematically exact method to infer the reaction rates [2, 10–12]. For demonstration and exemplification of the approach, we use three synthetic models which are commonly used in literature [9, 13–25], with similar details and parameters as used in the numerical simulation and further discuss in the following.

Discrete Dynamics. Gene Regulatory Networks comprise a collection of molecular regulators that interact with one another and various substances within the cell to control the expression levels of mRNA and proteins, ultimately determining the cell's function. Various mathematical models have been suggested, aiming to describe different aspects of the process. As described in the review [16], models can fall into three categories: logic, continuous, or discrete models. The latter primarily addresses interactions between individual molecules. The mathematical framework considered in the manuscript is thus from the third type - the discrete model described by multiple reactions and simulated using the Gillespie algorithm. These so-called single-molecule level models elucidate the stochasticity nature —which means that a given state can evolve into various distinct trajectories, each associated with a certain probability. Consequently, each reaction may have several outcomes, and we do not limit ourselves to a specific pathway.

(!) ASSUMPTION - the suggested approach assumes discrete stochastic dynamics, that can be described by the Master equation and simulated with the Gillespie algorithm.

Given Edge. The models used in the main text assume that the production rate of variable x_i; the rate of the event $x_i \to x_i + 1$, depends on the level of other molecules within the system, for example, the variable x_j. We note that the assumption specifies the existence of a given direct interaction between the two variables, x_i and x_j, denotes that one of the following conditions must be fulfilled; the production rate of x_i is func(x_j) or the production rate of x_j is func(x_i). The scope of this manuscript is limited to determining the direction of the interaction. Note that the function that specified the influence of one molecular level over the dynamics of the other, the function func(.), may remain unknown. Specifically, this function does not be from Michaelis-Menten or Hill-type function and these were chosen for demonstration as biology realistic influences only.

(!) ASSUMPTION - the interaction itself, i.e., the existence of an edge, is known.

Joint Measurements of (x_i, x_j)*.* As mentioned in the main text, the meaning of the variable x_i (variable x_j) is the level of individuals from type i (type j). In the main text, we concentrated on the interpretation that x_i is the number of individuals from the molecule type i, e.g., genes, mRNA, ribosome, or complex. However, there is no mathematical limitation that forces us not to interpret the variable x_i as the number of molecules from type X at a location i. This description is suitable for modeling transcription or translation complexes at a given location or codon. The inference approach requires the measurement of the joint probability $P(x_i, x_j)$ corresponding to the considered interpretation of each variable.

(!) ASSUMPTION - The two nodes under question are measured.

Coarse-Grain versus Detailed Models. Generally, the reactions model can be utilized to describe various processes, incorporating different aspects of each process. For example, models can account for multiple gene transcription and translation, feedback loops, ribosome stalling, or other possibilities. For analysis, one can simply "coarse-grain" these models, or leave them as complex as wanted. However, these changes in the model description may alter the assumed interaction framework and, consequently, the fundamental objective of the study, which is to determine the orientation of a specific edge. This is crucial because that edge may not exist in a different representation of the system, whether the model is simplified from a more complex description or made more intricate from a simpler one. As noted, the direction inference method operates on the premise that there is a known edge between two nodes, indicating an interaction between two variables.

(!) LIMITATION - A change in the assumption of the existence of an edge - either by coarse-graining of a complex model or vice-versa - changes the method's applicability since a core assumption is that the edge is given.

Michaelis-Menten Model. As previously said, the interactions' functional behavior defined in each of the models is not required to be from Michaelis-Menten or Hill-type function, and these were chosen for demonstration as realistic biological influences only. A simple description of the Michaelis-Menten kinetics, as commonly provided in the textbooks, is described in the following. In biochemistry, Michaelis–Menten kinetics describes enzyme-catalyzed reactions of substrate S and product P in the presence of the enzyme E. The reactions are typically described as $S + E \underset{k_-}{\overset{k_+}{\rightleftharpoons}} ES \overset{k_{\text{cat}}}{\rightarrow} E + P$. Assuming steady state $\partial_t[ES] = 0$ yields $[E][S]/[ES] = K_m$ where $K_m \equiv \frac{k_{\text{cat}}+k_-}{k_+}$ is the Michaelis-Menten constant. The conservation of the total enzyme concentration; $[E_{\text{total}}] = [E] + [ES]$ gives $\partial_t P = k_{cat}[E_{\text{total}}][S]/(K_m + [S]) \equiv V_m[S]/(K_m + [S])$. In [25] the authors note that the relation holds where the total enzyme concentration is low compared to the total substrate concentration, i.e. $[E_{\text{total}}] \ll [S_{\text{total}}] + K_m$.

The reaction can thus be written as $S \xrightarrow{\text{func}([S])} P$ with the influence of the concentration of the substrate S on the production of P is encapsulated in $\text{func}([S]) \equiv V_m[S]/(K_m + [S])$ where V_m is the maximal rate, and K_m is the concentration of S which provides half-maximum production rate of P.

Importantly, the 'coarse-grained' description of $S \xrightarrow{\text{func}([S])} P$ with $\text{func}([S]) \equiv V_m[S]/(K_m + [S])$ is not unique and can be derived from various multiple scenarios. For example, considering the first reaction is irreversible yields the same functional behavior with $K_m = k_{\text{cat}}/k_+$. Another derivation assumes chemical equilibrium such that $[E][S]/[ES] = k_+/k_- \equiv k_{\text{diss}} = K_m$ is the dissociation constant. Regardless of the detailed complex description that the model $S \xrightarrow{\text{func}([S])} P$ is based on, the assumption that the two variables S and P are linked, i.e, there is an edge between them, allows us to infer the interaction direction; whether $S \to P$ or $P \to S$ from their joint abundances snapshots.

[1] F. Edition, A. Papoulis, and S. U. Pillai, *Probability, random variables, and stochastic processes* (McGraw-Hill Europe: New York, NY, USA, 2002).

[2] T. Wittenstein, N. Leibovich, and A. Hilfinger, Quantifying biochemical reaction rates from static population variability within incompletely observed complex networks, PLOS Computational Biology **18**, e1010183 (2022).

[3] D. T. Gillespie, A general method for numerically simulating the stochastic time evolution of coupled chemical reactions, Journal of computational physics **22**, 403 (1976).

[4] R. C. Geary, The frequency distribution of the quotient of two normal variates, Journal of the Royal Statistical Society **93**, 442 (1930).

[5] D. V. Hinkley, On the ratio of two correlated normal random variables, Biometrika **56**, 635 (1969).

[6] I. Lestas, J. Paulsson, N. E. Ross, and G. Vinnicombe, Noise in gene regulatory networks, IEEE Transactions on Automatic Control **53**, 189 (2008).

[7] A. Marx and J. Vreeken, Telling cause from effect using mdl-based local and global regression, in *2017 IEEE international conference on data mining (ICDM)* (IEEE, 2017) pp. 307–316.

[8] P. Blöbaum, D. Janzing, T. Washio, S. Shimizu, and B. Schölkopf, Analysis of cause-effect inference by comparing regression errors, PeerJ Computer Science **5**, e169 (2019).

[9] M. Nitzan, J. Casadiego, and M. Timme, Revealing physical interaction networks from statistics of collective dynamics, Science advances **3**, e1600396 (2017).

[10] J. Schnakenberg, Network theory of microscopic and macroscopic behavior of master equation systems, Reviews of Modern physics **48**, 571 (1976).

[11] G. Haag, W. Weidlich, and P. Alber, Approximation methods for stationary solutions of discrete master equations, Zeitschrift für Physik B Condensed Matter **26**, 207 (1977).

[12] F. P. Kelly, *Reversibility and stochastic networks* (Cambridge University Press, 2011).

[13] M. Timme, Revealing network connectivity from response dynamics, Physical review letters **98**, 224101 (2007).

[14] D. L. Nelson, A. L. Lehninger, and M. M. Cox, *Lehninger principles of biochemistry* (Macmillan, 2008).

[15] S. Tripathi, D. A. Kessler, and H. Levine, Minimal frustration underlies the usefulness of incomplete regulatory network models in biology, Proceedings of the National Academy of Sciences **120**, e2216109120 (2023).

[16] G. Karlebach and R. Shamir, Modelling and analysis of gene regulatory networks, Nature reviews Molecular cell biology **9**, 770 (2008).

[17] B. C. Goodwin, Oscillatory behavior in enzymatic control processes, Advances in enzyme regulation **3**, 425 (1965).

[18] P. Ruoff, M. Vinsjevik, C. Monnerjahn, and L. Rensing, The goodwin oscillator: on the importance of degradation reactions in the circadian clock, Journal of biological rhythms **14**, 469 (1999).

[19] T. Schaffter, D. Marbach, and D. Floreano, Genenetweaver: in silico benchmark generation and performance profiling of network inference methods, Bioinformatics **27**, 2263 (2011).

[20] F. Liu, S.-W. Zhang, W.-F. Guo, Z.-G. Wei, and L. Chen, Inference of gene regulatory network based on local bayesian networks, PLoS computational biology **12**, e1005024 (2016).

[21] M. Foo, J. Kim, and D. G. Bates, Modelling and control of gene regulatory networks for perturbation mitigation, IEEE/ACM Transactions on Computational Biology and Bioinformatics **16**, 583 (2018).

[22] R. J. Prill, D. Marbach, J. Saez-Rodriguez, P. K. Sorger, L. G. Alexopoulos, X. Xue, N. D. Clarke, G. Altan-Bonnet, and G. Stolovitzky, Towards a rigorous assessment of systems biology models: the dream3 challenges, PloS one **5**, e9202 (2010).

[23] M. Ronen, R. Rosenberg, B. I. Shraiman, and U. Alon, Assigning numbers to the arrows: parameterizing a gene regulation network by using accurate expression kinetics, Proceedings of the national academy of sciences **99**, 10555 (2002).

[24] M. Santillán, On the use of the hill functions in mathematical models of gene regulatory networks, Mathematical Modelling of Natural Phenomena **3**, 85 (2008).

[25] J. K. Kim and J. J. Tyson, Misuse of the michaelis–menten rate law for protein interaction networks and its remedy, PLoS Computational Biology **16**, e1008258 (2020).
